# The Semi-Synthetic Peptide Lin-SB056-1 in Combination with EDTA Exerts Strong Antimicrobial and Antibiofilm Activity against *Pseudomonas aeruginosa* in Conditions Mimicking Cystic Fibrosis Sputum

**DOI:** 10.3390/ijms18091994

**Published:** 2017-09-16

**Authors:** Giuseppantonio Maisetta, Lucia Grassi, Semih Esin, Ilaria Serra, Mariano A. Scorciapino, Andrea C. Rinaldi, Giovanna Batoni

**Affiliations:** 1 Department of Translational Research and New Technologies in Medicine and Surgery, University of Pisa, Via S. Zeno 35-39, 56127 Pisa, Italy; gmaisetta@biomed.unipi.it (G.M.); lucia.grassi@med.unipi.it (L.G.); semih.esin@med.unipi.it (S.E.); 2 Department of Chemical and Geological Sciences, University of Cagliari, 09024 Monserrato, CA, Italy; ilaria.serra@unica.it; 3 Department of Biomedical Sciences, University of Cagliari, 09024 Monserrato, CA, Italy; scorciapino@unica.it (M.A.S.); rinaldi@unica.it (A.C.R.)

**Keywords:** *Pseudomonas aeruginosa*, cystic fibrosis, antimicrobial peptide, artificial sputum medium, biofilm, infrared spectroscopy

## Abstract

*Pseudomonas aeruginosa* is a major cause of chronic lung infections in cystic fibrosis (CF) patients. The ability of the bacterium to form biofilms and the presence of a thick and stagnant mucus in the airways of CF patients largely contribute to antibiotic therapy failure and demand for new antimicrobial agents able to act in the CF environment. The present study investigated the anti-*P. aeruginosa* activity of lin-SB056-1, a recently described semi-synthetic antimicrobial peptide, used alone and in combination with the cation chelator ethylenediaminetetraacetic acid (EDTA). Bactericidal assays were carried out in standard culture conditions and in an artificial sputum medium (ASM) closely resembling the CF environment. Peptide’s structure and interaction with large unilamellar vesicles in media with different ionic strengths were also investigated through infrared spectroscopy. Lin-SB056-1 demonstrated fast and strong bactericidal activity against both mucoid and non-mucoid strains of *P. aeruginosa* in planktonic form and, in combination with EDTA, caused significant reduction of the biomass of *P. aeruginosa* mature biofilms. In ASM, the peptide/EDTA combination exerted a strong bactericidal effect and inhibited the formation of biofilm-like structures of *P. aeruginosa*. Overall, the results obtained highlight the potential of the lin-SB056-1/EDTA combination for the treatment of *P. aeruginosa* lung infections in CF patients.

## 1. Introduction

Cystic fibrosis (CF) is a genetically inherited disease characterized by defects in the transport protein cystic fibrosis transmembrane conductance regulator (CFTR), causing the production of a sticky mucus that stands in the respiratory tract [1]. Consequently, CF patients display a very high susceptibility to chronic lung infections that represent an important cause of morbidity and mortality associated with the disease [2]. *Pseudomonas aeruginosa* is the leading cause of lung infections and a primary cause of death in CF patients [2]. During the course of the infection, adaptation of *P. aeruginosa* to lung environment involves the shift from a non-mucoid to a mucoid phenotype that has been associated with a worse clinical prognosis [3]. Conversion to a mucoid phenotype implies the overproduction of the polysaccharide alginate, which protects *P. aeruginosa* from the action of the currently available drugs and confers a selective advantage to the bacterium in the CF lung [3]. Moreover, a major factor contributing to the development of persistent infection is the formation of a biofilm, a well-known virulence factor of *P. aeruginosa* that renders bacteria recalcitrant to host immune response and antibiotic treatment. Traditionally, bacterial biofilms are referred to as surface-attached communities of bacteria encased within a self-produced extracellular matrix of complex composition [4]. It is now recognized that *P. aeruginosa* biofilms in the CF airway possess peculiar features. Instead of the classical surface-attached communities, detected in most biofilm infections, in the CF airway, *P. aeruginosa* forms biofilms composed of small clusters of planktonic bacterial aggregates containing dead neutrophils [5]. These non-attached cell aggregates, referred to as biofilm-like structures (BLS), appear to share many properties with conventional surface-attached biofilms, including dependence on quorum sensing, presence of subpopulations of dormant cells (“persisters”), and resistance to antibiotics [6].

The CF lung environment is characterized by the presence of abundant sputum that contains mucin, lipids, proteins, amino acids, ions and DNA released by dead neutrophils and promotes bacterial colonization [7,8]. Extracellular DNA contributes to render the sputum highly viscous by forming a gel-like matrix with other macromolecules that affects lung function and antibiotic efficacy. Once *P. aeruginosa* has established a chronic infection, it is hardly eradicated [9]. Therefore, there is a critical need for novel antimicrobial drugs that can effectively suppress bacterial infections in the challenging environment of the CF lung.

Over the last several decades, antimicrobial peptides (AMPs) have been intensely investigated as potential antibiotics against multidrug resistant bacteria [10]. Most AMPs are cationic molecules with an amphipathic structure as a consensus motif for antimicrobial activities that selectively target the membranes of bacteria via electrostatic forces. In contrast to standard antibiotics, AMPs are effective against both quiescent and actively growing bacteria, as they generally do not require metabolic processes for antimicrobial activity, display rapid killing kinetics, and demonstrate low propensity to select resistant mutants in vitro [11]. In recent years, the possible use of AMPs against microbial biofilms has been largely highlighted, as it appears that such molecules display several properties considered beneficial to prevent/eradicate biofilms [12,13,14].

We recently described an optimized semi-synthetic antimicrobial peptide called lin-SB056-1 [15]. Such a peptide has a β-type conformation and displays a perfect pattern of alternating hydrophilic and hydrophobic residues in its primary structure. This high intrinsic amphipathicity of the peptide induces a high structural stability of the peptide β-sheet oligomers on the membrane surfaces. These structural properties confer lin-SB056-1 a strong antimicrobial activity [16].

The biofilm mode of growth, together with lung mucus viscosity, reduces the effectiveness of conventional antibiotic therapy in CF [17]. Thus, the usage of adjuvants in CF therapy regimen has been proposed to improve the diffusion of antimicrobials [18]. Different studies have shown that the divalent cation chelator ethylenediaminetetraacetic acid (EDTA) can destabilize the biofilm structure by interfering with the ionic attractive forces among the biofilm matrix components [19,20,21]. Furthermore, EDTA is endowed with its own antimicrobial properties and has shown ability to potentiate the activity of conventional antibiotics against biofilms of a variety of clinical strains [22].

In the present study, we evaluated the antimicrobial and antibiofilm activity of the peptide lin-SB056-1, used alone and in combination with disodium-EDTA, in standard culture conditions as well as in an artificial sputum medium (ASM) that closely mimics the thick and stagnant airway mucus of CF patients. The structural determinants that drive the interaction of lin-SB056-1 with its target membranes were also explored. A rapid and strong bactericidal activity of the peptide/EDTA combination against planktonic cells as well as preformed biofilms of *P. aeruginosa* was demonstrated in standard conditions. When assayed in ASM, the peptide in combination with host-compatible concentrations of disodium-EDTA was able to kill both mucoid and non-mucoid strains of *P. aeruginosa* at relatively low concentrations and to almost completely inhibit the formation of BLS at non-hemolytic concentrations. Analysis of the modes of interaction of lin-SB056-1 with lipid vesicles mimicking bacterial membrane, performed using infrared spectroscopy as preferential biophysical technique, revealed that the peptide activity enhancement in the presence of EDTA appears not to be correlated with significant structural changes. This leads to hypothesize that the observed increased activity in the presence of EDTA might be due to the ability of such chelator to sequester membrane-associated divalent cations, thus probably synergizing with the peptide in perturbing membrane integrity. Overall, the results obtained suggest that the lin-SB056-1/EDTA combination may represent an attractive candidate to develop a new antimicrobial tool for the treatment of *P. aeruginosa* pulmonary infections in CF patients.

## 2. Results

### 2.1. Bactericidal Activity and Killing Kinetics of Lin-SB056-1 against Planktonic Cells of Mucoid and Non-Mucoid Strains of P. aeruginosa, in Sodium Phosphate Buffer (SPB) with 1% Tryptone Soya Broth (TSB)

To investigate the bactericidal activity of lin-SB056-1 against *P. aeruginosa*, minimal bactericidal concentrations (MBCs) of the peptide towards three non-mucoid and three mucoid strains were determined (Table 1). Remarkably, the peptide was bactericidal against all the tested strains at relatively low concentrations ranging between 1.56 and 3.12 μg/mL. Interestingly, the bactericidal concentrations against mucoid strains of *P. aeruginosa* were equal or even lower than those recorded against non-mucoid strains.

**Figure 1:**
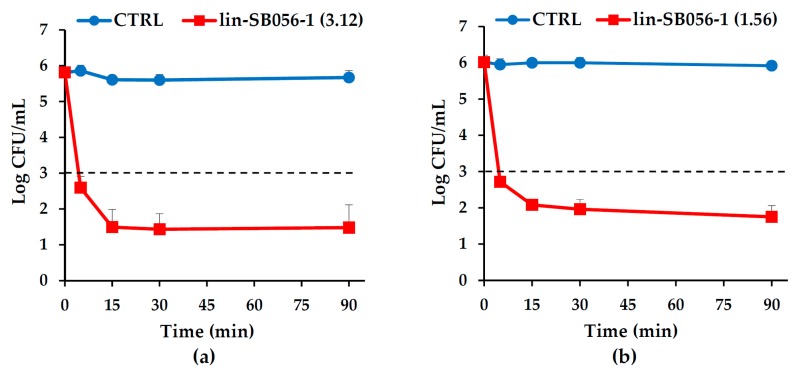
Time kill curves of lin-SB056-1 at its bactericidal concentrations against: (**a**) *P. aeruginosa* ATCC 27853; and (**b**) *P. aeruginosa* PaM01 in 10 mM Sodium Phosphate Buffer (SPB) at pH 7.4 supplemented with 1% Tryptone Soya Broth (TSB). The concentrations of lin-SB056-1 (reported in parenthesis) are expressed in μg/mL. Control (CTRL) represents bacteria incubated in the absence of the peptide. Bactericidal activity was defined as a reduction in the numbers of viable bacteria of ≥3-log colony forming units (CFU)/mL at any incubation time tested. The dotted lines represent three-log reduction in CFU number, corresponding to the definition of bactericidal activity. Data are reported as mean ± standard error of the mean of three independent experiments.

Next, killing kinetics of the peptide at its MBCs were performed against *P. aeruginosa* ATCC 27853 and PaM01, representative of non-mucoid and mucoid strains, respectively. As shown in Figure 1a,b, lin-SB056-1 exhibited very fast killing kinetics against both *P. aeruginosa* strains, reducing the bacterial inoculum by three-log within 5 min of incubation.

### 2.2. Synergistic Activity of Lin-SB056-1 in Combination with EDTA on Preformed Biofilms of Mucoid and Non-Mucoid Strains of P. aeruginosa

**Figure 2:**
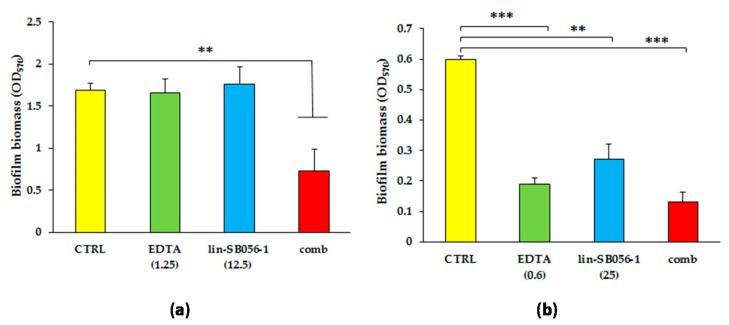
Activity of lin-SB056-1, used alone and in combination with EDTA, against preformed biofilms of *P. aeruginosa*: (**a**) ATCC 27853; and (**b**) PaM01 strain. The antibiofilm activity of the peptide, EDTA and peptide-EDTA combination was evaluated by crystal violet staining after 24 h of incubation. The concentrations of lin-SB056-1 and EDTA (reported in parenthesis) are expressed in μg/mL and mM, respectively. Control (CTRL) represents bacteria incubated in the absence of antimicrobial agents; comb: peptide/EDTA combination. Data are reported as mean ± standard error of the mean of three independent experiments. ** *p* < 0.01, *** *p* < 0.001 (one way analysis of variance [ANOVA] followed by Tukey–Kramer post-hoc test).

It is well recognized that the treatment of preformed biofilms is particularly challenging and that combinations of molecules targeting different biofilm components are needed to improve the therapeutic outcome [23]. Therefore, we tested the activity of lin-SB056-1 in combination with EDTA, a cation chelator known to destabilize the biofilm structure and favor the antimicrobial activity of conventional antibiotics as well as AMPs [22,24]. Established biofilms of two strains of *P. aeruginosa* (one non-mucoid and one mucoid) were exposed to various concentrations of lin-SB056-1 or EDTA, used alone or combined together, and the biofilm biomass was evaluated by crystal violet staining after 24 h. The results of the most powerful combinations are shown in Figure 2.

When lin-SB056-1 at 12.5 μg/mL and EDTA at 1.25 mM were used alone, no effect on mature biofilms of the non-mucoid strain *P. aeruginosa* ATCC 27853 was observed (Figure 2a). In contrast, the combinations of the two agents caused a reduction of more than 50% of the biofilm biomass of the same strain, as compared to the control biofilm. Similar results were obtained when the peptide was used at 25 μg/mL. The peptide used alone at 25 μg/mL was quite active against mature biofilms of the mucoid strain PaM01, reducing the biofilm biomass of such strain by 55% after 24 h incubation (Figure 2b). In agreement with the dependence on calcium to produce appreciable amount of biofilm by the mucoid strain PaM01 (see Section 4.5), established biofilms of such strain were also highly susceptible to relatively low concentrations of EDTA (0.6 mM, Figure 2b). Finally, when the peptide (25 μg/mL) and EDTA (0.6 mM) were combined together, the reduction of biofilm biomass of the PaM01 strain reached 80% as compared to the untreated control.

### 2.3. Bactericidal Effect of Lin-SB056-1 in Combination with EDTA against Planktonic P. aeruginosa in Artificial Sputum Medium (ASM)

**Figure 3:**
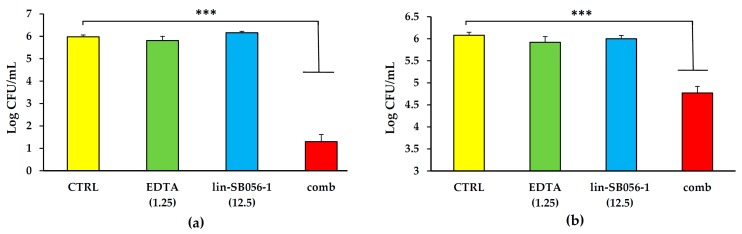
Bactericidal activity of lin-SB056-1 used alone and in combination with EDTA against: (**a**) *P. aeruginosa* ATCC 27853; and (**b**) *P. aeruginosa* PaM01 strain in ASM 80% after 1.5 h of incubation. The concentrations of lin-SB056-1 and EDTA (reported in parenthesis) are expressed in μg/mL and mM, respectively. Control (CTRL) represents bacteria incubated in the absence of antimicrobial agents; comb: peptide/EDTA combination. Data are reported as mean ± standard error of the mean of three independent experiments. *** *p* <0.001 (one way ANOVA followed by Tukey–Kramer post-hoc test).

The accumulation of a thick and stagnant mucus in the airways of CF patients provides a suitable environment for the persistence of *P. aeruginosa* within the lung of such patients. Thus, to explore the peptide’s potential to exert anti-*Pseudomonas* activity in such environment, we tested the bactericidal activity of lin-SB056-1 against *P. aeruginosa* ATCC 27853 and the mucoid strain PaM01 in ASM that closely mimics the composition of CF sputum. Although lin-SB056-1 used at 12.5 μg/mL was inactive in ASM 80% against both the ATCC 27853 and the PaM01 strains, the peptide showed a strong synergistic effect when used in combination with 1.25 mM EDTA (Figure 3). In particular, the peptide/EDTA combination caused a reduction of four-log of the colony forming units (CFU) number of the ATCC strain (*p* < 0.001, Figure 3a), while, when tested against the mucoid strain PaM01, the combination reduced the bacterial inoculum by 1.5-log (*p* < 0.001, Figure 3b).

Bactericidal assays were also performed at 24 h against the ATCC 27853 strain. The results obtained (Figure S1) showed that at such incubation time the combination was also able to cause a statistically significant reduction of CFUs (approximately two-log) as compared to EDTA and lin-SB056-1 used alone.

Finally, to get further insights on the activity of lin-SB056-1 in in vivo-like conditions, bactericidal assays were performed in the presence of 12.5% serum, as it may represent a component of the lung microenvironment during chronic infection/inflammation. As shown in Figure S2, lin-SB056-1, at the concentration of 12.5 μg/mL, exerted a very strong bactericidal effect against the ATCC 27853 strain at both 1.5 and 24 h.

### 2.4. Activity of Lin-SB056-1 in Combination with EDTA in Preventing the Formation of Biofilm-Like Structures (BLSs) of P. aeruginosa ATCC 27853 in ASM 

**Figure 4:**
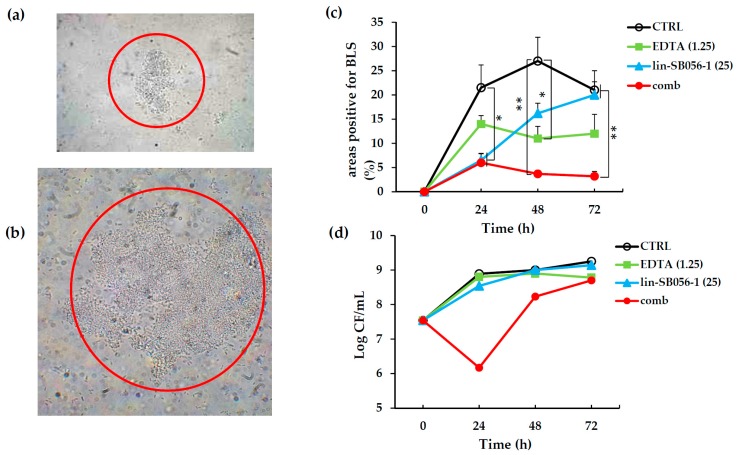
Visualization of a BLS under light microscopy at: (**a**) 400×; and (**b**) 1000× magnification, after 72 h of incubation; (**c**) Kinetics of lin-SB056-1 activity, used alone and in combination with EDTA, on the formation of BLSs of *P. aeruginosa* ATCC 27853 in ASM 80%. Data are reported as mean ± standard error of the mean of three independent experiments. * *p* < 0.05, ** *p* < 0.01 (one way ANOVA followed by Tukey–Kramer post-hoc test); (**d**) Killing kinetics of activity of lin-SB056-1, used alone and in combination with EDTA, against *P. aeruginosa* ATCC 27853 in ASM 80%. Data from a representative experiment are shown. The concentrations of lin-SB056-1 and EDTA (reported in parenthesis) are expressed in μg/mL and mM, respectively. Control (CTRL) represents bacteria incubated in the absence of antimicrobial agents; comb: peptide/EDTA combination.

It has been previously reported that *P. aeruginosa* growth in ASM resembles bacterial growth during lung infection of CF patients, with the formation of self-aggregating BLS [8]. To obtain BLS, *P. aeruginosa* ATCC 27853 was cultured in ASM in microaerophilic atmosphere and in static conditions for 72 h. An almost two-log increase in the number of *P. aeruginosa* cells was observed at the end of the incubation period as compared to the inoculum, demonstrating that the artificial medium was able to support bacterial growth (CTRL, Figure 4d). Furthermore, the conditions adopted also allowed *P. aeruginosa* to form cell-aggregates easily visible under light microscope at 400× or 1000× magnification (Figure 4a,b). Using this experimental system, we evaluated the ability of the lin-SB056-1/EDTA combination to inhibit the formation of BLSs over the time. As shown in Figure 4c, the peptide used at 12.5 μg/mL in combination with 1.25 mM EDTA was able to almost completely inhibit the formation of BLS for the full length of the experiment (72 h). The number of viable *P. aeruginosa* cells cultivated in ASM was also assessed at time 0 and at 1-day intervals in the presence of lin-SB056-1, EDTA or both components. As shown in Figure 4d, a marked reduction in bacterial cell number (almost three-log as compared to the untreated control) was observed at 24 h in the presence of the peptide/EDTA combination. At later time points, the viable count progressively increased, reaching levels similar to the control, after 72 h of incubation. No evident effect on CFU number was observed when EDTA and lin-SB056-1 were used alone.

### 2.5. Interaction of Lin-SB056-1 with Lipid Vesicles in the Presence and Absence of EDTA, as Observed through Infrared Spectroscopy

**Figure 5:**
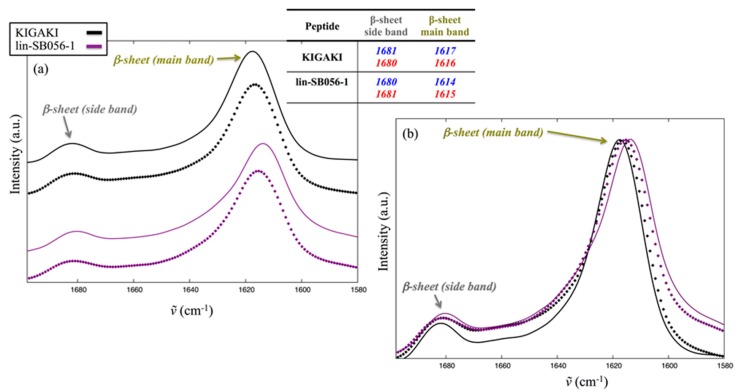
(**a**) Stacking plot of the normalized amide I band from RF samples in PBS of KIGAKI (black) and lin-SB056-1 (purple); (**b**) Superposition of the same spectral region reported in (**a**). Spectra acquired in the presence of LUVs with 25 and 75%_mol_ 1-palmitoyl-2-oleoyl-*sn*-glycero- 3-phospho-(1’-*rac*-glycerol) (POPG) are shown with solid and dotted lines, respectively. The table reports the position of the two components characteristic of β-sheets, with data pertaining at the 25 and 75%_mol_ POPG reported in blue and red, respectively.

Figure 5 shows the amide I band from spectra collected on rehydrated film (RF) samples (see Section 4.3). This is centered at ~1650 cm^−1^ and, being sensitive to backbone conformation and rigidity while scarcely influenced by the side chains [25,26], bears important information on peptide secondary structure [27,28]. Amide I of KIGAKI, whose sequence is [KIGAKI]_3_ and was employed as reference for β-sheets, is clearly dominated by two components (Figure 5): the most intense component below 1620 cm^−1^ and the so-called secondary one at ~1680 cm^−1^ are characteristic for β-sheets formation, in agreement with the literature on this model peptide [29]. The large separation between the two amide I components is typical of antiparallel β-sheets [27,28]. The amide I band profile of lin-SB056-1 (Figure 5) was comparable to that of KIGAKI, with only slight differences in the position of the main component. On the other hand, significant differences were observed on the low wavenumber side of the amide I (1590–1580 cm^−1^), where the significantly higher absorption in the case of lin-SB056-1 is mostly due to guanidine group from arginine side chains, absent in KIGAKI.

**Figure 6:**
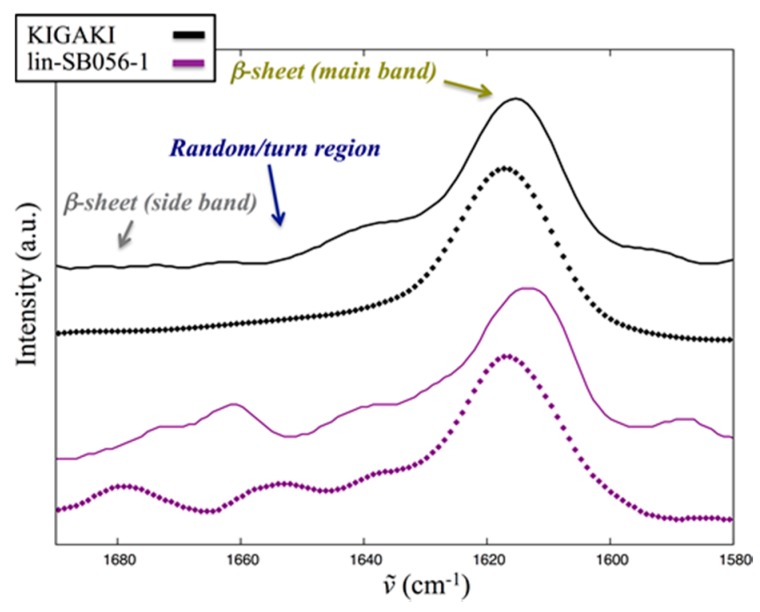
Stacking plot of the normalized amide I band from FH samples in PBS of KIGAKI (black) and lin-SB056-1 (purple). Spectra acquired in the presence of LUVs with 25 and 75%_mol._ POPG are shown with solid and dotted lines, respectively.

Signal/noise (S/N) ratio is usually good in RF spectra. However, whenever possible, it is advisable to also inspect the corresponding spectra acquired from fully hydrated (FH) samples that represent the more physiological condition of fully hydration [28,30,31,32]. Figure 6 shows the results for both lin-SB056-1 and KIGAKI. Despite the lower S/N ratio, FH spectra confirm that lin-SB056-1 forms β-sheets on top of both of the differently charged vesicles used in the present study. When compared to RF spectra, a higher contribution from both random and turn components is clearly visible in the amide I band between 1660 and 1640 cm^−1^, despite β-sheets components still dominate the amide I band. The proportion of such unordered contributions is higher in lin-SB056-1 than KIGAKI. Interestingly, both peptides showed a significant increase of structure order when moving from 25 to 75%_mol._ 1-palmitoyl-2-oleoyl-sn-glycero- 3-phospho-(1’-rac-glycerol) (POPG) vesicles (unordered components decreased in relative intensity, Figure 6).

**Figure 7:**
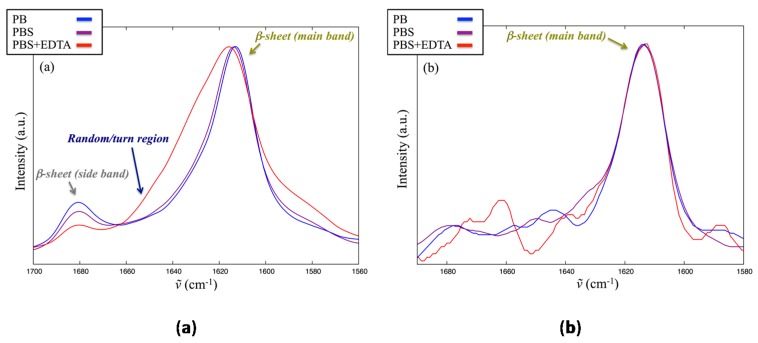
Superposition of the normalized amide I band from: (**a**) RF; and (**b**) FH samples of lin-SB056-1 in three different media, namely, PB (blue), PBS (purple) and PBSE (red). LUVs with 25%_mol_. POPG were used.

To investigate the influence of ionic strength on structure order, or the effect of possible direct coordination of the peptide’s positive groups, lin-SB056-1 was also analyzed in the presence of 25%_mol_ POPG vesicles but using PB (low ionic strength) or PBS supplemented with EDTA instead of the standard PBS. Figure 7 shows the amide I region of the infrared spectrum acquired both from RF and FH samples. No significant differences are observed between spectra acquired in PBS and PB. In the presence of EDTA, a significant proportion of random/turn contribution is observed in the RF spectrum (Figure 7a), which is also visible (although to a lesser grade) in the FH spectrum despite the higher S/N (Figure 7b). In addition, both spectra show a difference in the guanidine group vibration (1590–1580 cm^−1^). These results suggest that EDTA interacts with peptide positive side chains and disturbs the formation of regular and extended β-sheets, at least in RF samples. However, in the corresponding FH samples, which reflect a more physiological condition, differences with respect to the spectrum acquired in the absence of EDTA are less pronounced, and overall the observed spectrum is still compatible with a β-sheet structure (Figure 7b).

## 3. Discussion

The development of resistance to currently available antibiotics by pathogens involved in respiratory infections of CF patients is an issue causing increasing concern [33]. In particular, it is reported that about one-half of patients with CF are stably colonized with multidrug-resistant (MDR) *P. aeruginosa* [34]. To date, advancements in antimicrobial treatments of chronic *P. aeruginosa* infections in CF patients mainly rely on the use of aerosolized antibiotic formulations [35]. Although inhalation of antibiotics allows reaching high concentrations of the drugs directly in the lung, antimicrobial resistance can still develop as an inevitable consequence of the repeated antibiotic use needed to treat acute exacerbations or to lower the bacterial burden of chronically colonized patients [35]. As novel anti-Gram-negative antibiotics are scarce in the developmental pipeline and the therapeutic options for multidrug-resistant *P. aeruginosa* infections are limited, the identification and pre-clinical testing of new molecules with therapeutic potential is of paramount importance.

In this scenario, the interest in the use of AMPs as antimicrobials for the treatment of CF infections has increased considerably and many different AMPs have been tested in in vitro and in vivo models of CF infections with promising results [36,37,38]. As AMP activity against bacterial membranes is less specific than that of many conventional antibiotics, the risk of developing bacterial resistance to AMPs is considered low even after prolonged therapies. In addition, AMPs are often active against MDR strains and, unlike other peptide-based antibiotics currently in clinical use (e.g., colistin and daptomycin), exhibit a broad-spectrum of antibacterial activity. This latter property could be favorable for the treatment of pulmonary mixed infections that often occur in CF patients. Furthermore, although often highly inhibited in biological fluids [39], AMPs can be optimized by chemical modifications that improve their stability and activity and lower their toxic potential in physiological conditions [15,40].

In this regard, we have previously described a semi-synthetic AMP, SB056, identified by selecting a random phage library against whole *Escherichia coli* cells [41]. An analog of SB056 with improved amphipathic profile, lin-SB056-1 (KWKIRVRLSA), was obtained by exchanging the position of the first two residues of the original sequence but leaving the rest absolutely unchanged, net positive charge included. This simple modification proved to have deep consequences on the biological properties of the optimized peptide, which displayed significantly stronger activity against both Gram-positive and Gram-negative bacteria, in particular when tested in broth supplemented with physiological concentration of electrolytes [15,16].

In the first part of the present study, we demonstrated that lin-SB056-1 is highly active against both mucoid and non-mucoid *P. aeruginosa* strains, irrespective of their resistance phenotype. The shift towards a mucoid phenotype and the consequent overproduction of alginate is regarded to play a pivotal role in chronic *P. aeruginosa* infections, as alginate renders *P. aeruginosa* strains more tolerant to antibiotics [42]. Importantly, we found that the bactericidal concentrations of lin-SB056-1 against the mucoid strains of *P. aeruginosa* were similar or even lower than those recorded against non-mucoid strains. This suggests that the peptide is able to penetrate the alginate barrier and interact with its bacterial target(s). The peptide was not only bactericidal at relatively low concentrations, but also showed a very rapid killing effect (within 5 min) towards representative non-mucoid and mucoid strains of *P. aeruginosa*. This rapid killing time is compatible with a membrane-perturbing mechanism of action, in agreement with previous observations showing that the formation of extended β-sheets leads to an enhanced antimicrobial activity of lin-SB056-1 causing a marked alteration of membrane integrity [16].

Infrared spectroscopy analysis of the interaction of lin-SB056-1 with lipid vesicles mimicking bacterial membrane confirmed the tendency of the peptide to form β-sheets on top of the charged vesicles used in the present study, with an increase in structure order as the experimental system shifted from a lower to a higher percentage of charged lipids. The formation of β-sheets was also verified by comparison with the behavior of the well-characterized model peptide KIGAKI, and was retained even at physiological salt concentration (PBS). In the presence of EDTA, recorded RF spectrum indicates that EDTA interacts with peptide positive side chains and disturbs the formation of regular and extended β-sheets. However, in the FH spectrum, which reflects a more physiological condition, differences with respect to the spectrum acquired in the absence of EDTA are less pronounced, and overall the observed spectrum is still compatible with a β-sheet structure. Thus, based on what was experimentally observed, it might be concluded that EDTA does not modulate the structural properties of lin-SB056-1 in a significant manner, with the peptide killing bacteria through the formation of β-sheet on the membrane surface. The observed increased activity of the peptide in the presence of EDTA might be due to the ability of such chelator to sequester membrane-associated divalent cations, probably synergizing with the peptide in perturbing membrane integrity and thus hastening bacterial demise. Another possibility can be envisaged. Given its inherent characteristics, infrared spectroscopy forces one to work at high peptide/lipid ratio. It is possible that at lower peptide/lipid ratio, a condition closer to reality, the effects of EDTA on the structural order of lin-SB056-1 could be considerably stronger, possibly preventing the formation of extended β-sheets. This could be no detrimental to peptide’s antimicrobial activity. On the contrary, it might help it, in analogy to what has been shown in the case of Tachyplesin-I, a natural arginine-rich β-hairpin antimicrobial peptide for which the formation of extended structures was considered responsible for the loss of peptide in-plane mobility and consequently the reduction in peptide activity [43]. In the case of biofilms, this EDTA-peptide interaction could even be relevant in favoring peptide’s mobility through the extracellular matrix.

It is well established that the preferred mode of growth of *P. aeruginosa* in CF lung is as biofilm, a complex community that provides bacteria with a protective environment from the immune system and antimicrobial treatment. At present, our weapons against biofilms are very limited and rely on early aggressive treatment to prevent biofilm formation or administration of high doses of systemic/local antibiotics in combinations to eradicate preformed biofilms. Once colonization with *P. aeruginosa* has occurred and the organism has switched to the biofilm mode of growth, it is rare that it is eradicated from the respiratory tract of patients with CF. Many AMPs have proven good activity against a number of CF pathogens grown as biofilms [12]. Indeed, AMPs display several properties of an “ideal” anti-biofilm agent including their activity against metabolically inactive or poorly active cells that are abundant in biofilms, overcoming the limit of many traditional antibiotics that require actively growing bacterial cells to act [13].

We have previously reported that the optimized lin-SB056-1 peptide is highly active in preventing in vitro the formation of biofilms by *P. aeruginosa* [15]. In the present study, we sought to assess the peptide’s activity against preformed biofilms (24 h-old) that are known to be much more refractory than forming biofilms to treatment with antimicrobials including conventional antibiotics and AMPs. In the case of AMPs, this is mainly attributed to the multiple interactions that they may establish with the components of the biofilm extracellular matrix such as extracellular DNA, proteins and polysaccharides [13]. These interactions may reduce the bioavailability of the peptides and lower their anti-biofilm potential. In particular, *P. aeruginosa* secretes three types of exopolysaccharides, Pel, Psl, and alginate, all of which play a role in the tolerance to antimicrobials when bacteria grow as aggregates [44,45]. Thus, the use of disassembling agents is considered a good strategy to enhance the therapeutic potential of AMPs and other antimicrobials against mature biofilms. A number of compounds including lytic enzymes, furanones, triterpenes and D-amino acids have shown ability to disassemble the biofilm structure [46,47,48,49]. In this study, we used as potentiating agent the cation chelator EDTA. Unlike many other disassembling agents, EDTA is currently clinically approved for a number of medical treatments including the therapy of lead poisoning, heavy metal detoxification and hypercalcemia [19,50]. It has also proved to be safe and highly active when used in conjunction with antifungal agents in a rodent model of invasive pulmonary aspergillosis [51] and to markedly reduce the minimum inhibitory concentration of conventional antibiotics in a mouse model of lung infection with mucoid *P. aeruginosa* [21]. When sub-inhibitory concentrations of lin-SB056-1 and EDTA were combined together a net synergistic effect in the reduction of biofilm biomass of the non-mucoid *P. aeruginosa* ATCC 27853 strain was observed. In agreement with previous studies, the observed synergistic effect suggests that EDTA may lead to the breakdown of the biofilm by chelating divalent metal ions, such as iron, calcium and magnesium, which have an important role in stabilizing the biofilm extracellular matrix [19,20,21]. Such disassembling effect of EDTA may facilitate the diffusion of the peptide through the biofilm layers. Interestingly, when EDTA was used alone against mature biofilms of the mucoid strain PaM01, it exerted a highly significant antibiofilm activity at relatively low concentrations and produced an additive effect in combination with the lin-SB056-1 peptide. The susceptibility of PaM01 biofilms to EDTA is in agreement with previous studies that demonstrated the ability of calcium not only to increase the thickness of biofilms of a mucoid strain of *P. aeruginosa*, but also to play regulatory roles in bacterial gene expression [52]. For instance, calcium addition was reported to cause an eight-fold increase in *alg* gene expression in biofilms of a mucoid strain of *P. aeruginosa*, and to induce the expression of other relevant bacterial virulence factors suggesting that the chelating effect of EDTA may act at multiple levels against biofilms of mucoid strains of *P. aeruginosa*. Furthermore, it has been demonstrated that the viscosity of alginate is determined largely by its affinity for divalent cations, particularly calcium, which control the degree of cross-linking [18]. Thus, EDTA may increase the diffusion rates of lin-SB056-1 through the biofilm matrix by reducing alginate viscosity.

An important aspect of the preclinical testing of AMPs as antimicrobial/antibiofilm agents is the assessment of their activity in in vivo-like conditions as it is known that many AMPs are highly or totally inhibited in such conditions. In particular, the CF airway environment is very selective due to the presence of high concentrations of salts, mucin and DNA that may interfere with the antimicrobial action of AMPs [53,54]. Therefore, to explore further the therapeutic potential of the lin-SB056-1-EDTA combination, bactericidal assays were carried out in a previously described ASM that closely mimics the CF sputum [5]. In this very complex medium, a strong synergistic bactericidal effect of the combination was still observed against both the mucoid and non-mucoid strains of planktonic *P. aeruginosa*. It is well known that EDTA has a detrimental effect on the outer membrane permeability of Gram-negative bacteria [20]. By displacing divalent cations from their binding sites in the lipopolysaccharide (LPS), EDTA may cause leakage of LPS, thus increasing the permeability to lin-SB056-1 molecules and their interaction with the bacterial membranes. In addition, at least part of the synergistic effect observed in ASM could be ascribed to the ability of EDTA to promote the diffusion of the peptide in ASM, by reducing the viscosity of the sputum. This hypothesis is supported by a recent study by Gustafsson and coworkers who demonstrated that CF intestinal mucus can be transformed to an almost normal mucus in the presence of EDTA [55]. In particular, it has been reported that at acidic pH and in the presence of calcium, the type II-mucin forms large aggregates that are dissolved by removing calcium ions [55]. Thus, the chelating effect of EDTA could contribute to reduce the viscosity of the ASM used in this study, which possesses a mild acidic pH (6.8) and contains calcium at a concentration of approximately 10 mM as an element of casamino hydrolysate used as a source of amino acids [56].

In chronically infected lungs of CF patients, *P. aeruginosa* persists in the form of antibiotic-tolerant bacterial aggregates floating in the airways mucus and exhibiting a phenotype closely resembling that of surface-attached biofilms [57]. It has been demonstrated that in microaerobic conditions ASM supports the formation of BLSs similar to the bacterial aggregates described within the CF airways [5,8,54]. Thus, we sought to investigate the effect of our peptide/EDTA combination on the formation of BLSs by the *P. aeruginosa* ATCC 27853 strain in ASM. Interestingly, an almost total inhibition of BLSs formation was observed during the entire observational period (three days). In the first 24 h of incubation, inhibition of BLSs formation correlated with a sharp reduction in viable count. As high inoculum (10^7^–10^8^ CFU/mL) of stationary phase bacteria are needed to allow BLSs formation in ASM [5,58], the killing effect exerted by the combination at 24 h may have contributed to keep the cell density at low levels, impeding BLSs formation at this time point. At later time points, a progressive increase in cell number was observed suggesting an administration regiment every 24 h. Nevertheless, despite the increment in cell number, the BLS count remained low up to 72 h, suggesting that the lin-SB056-1 peptide and/or EDTA may somehow interfere with the phenomenon of cell aggregation, by directly interacting with bacterial cells and/or by sequestering medium components essential for biofilm formation [59].

Despite our attempts, we were not able to obtain BLSs of the mucoid strain PaM01 in ASM in agreement with previous reports that demonstrated that *P. aeruginosa* strains may greatly differ in their ability to produce BLSs in such a medium [5]. Nevertheless, the results obtained with the ATCC 27853 strain suggest that the lin-SB056-1/EDTA combination may impede the formation of BLSs in conditions very relevant to the CF environment. As BLSs formation is believed one of the major obstacles to the success of the therapy, ability of the combination to maintain bacterial cells in a planktonic status could render them more susceptible to the action of antimicrobial drugs.

It is noteworthy that both lin-SB056-1 and EDTA at the concentrations used in this study (12.5/25 μg/mL lin-SB056-1, and 1.25 mM EDTA) did not result hemolytic towards human erythrocytes in previous studies [15,60] further highlighting the therapeutic potential of the peptide.

## 4. Materials and Methods

### 4.1. Pseudomonas aeruginosa Strains and Culture Conditions

*P. aeruginosa* strains were isolated from sputum of CF patients at the Microbiology Unit of the University Hospital of Pisa, Italy. MALDI-TOF (Bruker Daltonics, Bremen, Germany) was used for the identification of the bacterial species and VITEK^®^ 2 system (bioMérieux, Bagno a Ripoli, Italy) for the definition of the antimicrobial susceptibility profile, according to The European Committee on Antimicrobial Susceptibility Testing (EUCAST Clinical Breakpoints http://http://www.eucast.org/clinical_breakpoints/). Five clinical isolates of *P. aeruginosa* (PaNM01, PaNM02, PaM01, PaM02, and PaM03) and the reference strain *P. aeruginosa* ATCC 27853 were used in the study. *P. aeruginosa* strains were qualitatively evaluated regarding their ability to express a mucoid phenotype. To this aim, bacterial suspensions were streaked onto the surface of MacConkey agar (bioMérieux, Marcy-l′Étoile, France) and Cetrimide agar (Sigma-Aldrich, St. Louis, MO, USA) plates and incubated at 37 °C for 48 h. The development of colonies with a slimy phenotype was rated as positive for the presence of mucoid, alginate-producing cells. The strains were grown with shaking in TSB (Oxoid, Basingstoke, UK) or in Luria Bertani broth (LB) at 37 °C for liquid cultures and on Tryptone soy agar (TSA) (Oxoid, Hampshire, UK) for 48 h at 37 °C for CFU determination.

### 4.2. Peptide and EDTA Solutions

Lin-SB056-1 peptide (KWKIRVRLSA) was purchased from Peptide Protein Research, Ltd. (Fareham, UK) with a purity of 98%; it was provided as chloride salt and used without further purification. Peptide [KIGAKI]_3_, HCl salt, 95% purity with C-terminus amidated was kindly provided by Prof. Anne S. Ulrich and Dr. Parvesh Wadhwani from the Karlsruhe Institute of Technology (Karlsruhe, Germany). EDTA was obtained from Sigma-Aldrich. A stock EDTA solution (0.5 M) was prepared in milli-Q water and the pH of the solution was adjusted to 8.0 with NaOH. The stock solution was then diluted in milli-Q water to obtain a working solution of 50 mM that was sterilized by filtration and stored at 4 °C.

### 4.3. Infrared Spectroscopy

Lin-SB056-1 was studied in three different media with D_2_O as solvent, namely, (i) phosphate buffer (PB; potassium H_2_PO_4_/HPO_4_; 10 mM total phosphates; pH 7.4); (ii) phosphate buffer saline (PBS; prepared from PB with 150 mM NaCl) and (iii) PBS with Na-EDTA (PBSE; prepared from PBS with [EDTA]/[peptide] molar ratio of 3:1). Peptide was dissolved at 2.0 mM in the presence of 20 mM large unilamellar vesicles (LUVs). The latter were prepared from a mixture of 1-palmitoyl-2-oleoyl-*sn*-glycero-3-phosphatidylcholine (POPC) and POPG by the extrusion method, as described in details elsewhere [61]. The [POPC]/[POPG] molar ratio was always 3:1, but, in the case of PBS, experiments were also performed with LUVs prepared with [POPC]/[POPG] ratio of 1:3. Model peptide KIGAKI (whose sequence is [KIGAKI]_3_) was employed as reference for β-sheets [29]. All infrared spectra were acquired with a Bruker Vector-22 equipped with a diamond single-reflection Attenuated Total Reflection accessory (Platinum ATR module) and a liquid nitrogen cooled MCT detector. The software OPUS (version 6.5, Bruker) was used for both spectra acquisition and data analysis. Spectra were acquired from 10 μL of the sample deposited on the ATR crystal. These FH samples were acquired in the 3500–600 cm^−1^ spectral range, with 4 cm^−1^ resolution, by applying automatic atmosphere compensation and 32 scans. Background of the clean ATR crystal was acquired with the same parameters immediately before each measurement. Spectrum of buffers was also acquired in the same experimental conditions to be subtracted from samples’ spectra. Spectra were also recorded from the same samples as RF, obtained by depositing 10 μL of the dispersion on the ATR crystal and by gently evaporating the solvent under a stream of nitrogen. Rehydration was achieved by placing a small Petri dish over the film after deposition of several drops of D_2_O around the crystal. Spectra acquisition was started after one minute. Acquisition parameters were the same as for FH samples but 16 scans were acquired for the RF ones. The spectrum of the corresponding LUVs rehydrated film was acquired to be used for both medium and vesicles signals removal.

### 4.4. Bactericidal Activity in Sodium-Phosphate Buffer (SPB)

The bactericidal activity of lin-SB056-1 against *P. aeruginosa* strains was evaluated in sodium-phosphate buffer (SPB) 10 mM, pH 7.4 supplemented with 1% TSB (SPB/TSB) by the microdilution method. To this aim, bacterial strains were grown in TSB until exponential phase, washed by centrifugation and resuspended in SPB to reach a density of 1 × 10^7^ CFU/mL. Bacteria, contained in a volume of 10 μL, were added to 90 μL of SPB/TSB containing the peptides at different concentrations (ranging from 0.78 to 12.5 μg/mL). To check for cell viability, samples containing bacteria suspended in SPB/TSB in the absence of the peptide were established. After an incubation at 37 °C with shaking for various time intervals (5, 15, 30, and 90 min), the samples were diluted 10-fold in TSB and plated on TSA for CFU counting. The MBC was defined as the minimal concentration of the peptide causing a reduction of at least 3-log in the number of CFUs after 90 min of incubation. In a set of experiments, peptide’s activity was assessed at 1.5 and 24 h in the presence of serum by adding heat inactivated pooled human serum, obtained from 6 healthy blood donors, to the assay mixture to reach a final serum concentration of 12.5%.

### 4.5. Biofilm Treatment Assays 

*P. aeruginosa* ATCC 27853 and the mucoid strain PaM01 were cultured overnight in TSB at 37 °C with agitation. After diluting bacterial suspensions 1:100 in TSB, aliquots of 100 μL were seeded into wells of flat-bottom polystyrene 96-well microtiter plates (Corning Costar, Lowell, MA, USA). Biofilms were left to form for 24 h (ATCC 27853 strain) or 7 days (PaM01 strain) at 37 °C in the absence of antimicrobial compounds. Previously it has been demonstrated that mucoid strains of *P. aeruginosa* need a supplement of calcium to produce appreciable amount of biofilm in vitro [52]. In agreement with this observation, in our hands the PaM01 strain was unable to form biofilm in the absence of calcium. Therefore, the medium of this strain was enriched with 0.5 mM calcium chloride to allow the formation of biofilms. Established biofilms were then washed three times with SPB in order to remove non-adherent cells, and exposed in fresh medium (TSB diluted 1:1 with SPB) to different concentrations of lin-SB056-1 (from 6.25 to 50 μg/mL) alone and in combination with EDTA (from 0.3 to 2.5 mM). Wells added with EDTA only were also established. In the case of the strain PaM01, the medium was enriched with 0.25 mM calcium chloride. Microplates were incubated statically at 37 °C for 24 h. Following incubation, the biofilm biomass was determined by crystal violet staining. After 24 h incubation bacterial biofilms were washed twice with 200 μL of PBS and incubated at 60 °C for 60 min to dry. The wells were then stained at room temperature with 150 μL of 1% crystal violet in 7% ethyl alcohol solution for 10 min. The plates were then washed with 200 μL of sterile distilled water and air-dried at 37 °C for 30 min. To dissolve the dye associated with attached biofilm, 200 μL of 30% acetic acid were added to the wells and the OD_570_ absorbance was measured on a microplate reader (Bio-Rad Laboratories, Segrate, Italy).

### 4.6. Bactericidal Assays in ASM

For ASM preparation, the components were dissolved into sterile water at the following concentrations: mucin (Sigma-Aldrich), 0.5% (*w*/*v*); unsheared salmon sperm DNA (Sigma-Aldrich), 0.4% (*w*/*v*); NaCl, 0.5% (*w*/*v*); KCl, 0.2% (*w*/*v*); casamino acids (Oxoid), 0.5% (*w*/*v*); egg yolk emulsion (0.15%, source of lecithin; sterile; Sigma-Aldrich). After each addition, the mixture was vigorously vortexed. The pH of the solution was adjusted at 6.8 with Tris-HCl 1 M, pH 8.5 [5]. *P. aeruginosa* was grown overnight in LB broth, pelleted, washed with PBS, and resuspended in ASM at a density of 10^6^ CFU/mL. A volume of 10 μL of the peptide (3.12 to 25 μg/mL) and/or 10 μL of EDTA (0.62 to 2.5 mM) were added to 80 μL of ASM containing bacteria. To check for cell viability, bacteria were suspended in ASM 80% alone. After an incubation at 37 °C with shaking for 90 min (or 24 h), the samples were diluted 10-fold in TSB and plated on TSA, to determine the number of viable cells.

### 4.7 Static Culture System for the Development of Biofilm-Like Structures (BLSs) in ASM

For the development of BLSs, *P. aeruginosa* ATCC 27853 was grown overnight in LB broth and then diluted into ASM to a final cell density of 10^7^ CFU/mL. Bacteria were dispensed into 96-well microplate in the presence of lin-SB056-1 or EDTA alone or in combination. The plates were incubated at 37 °C in static (non-shaking) conditions under microaerophilic conditions for different time intervals before assessing BLS formation. To this aim, 40 μL of each bacterial suspension was gently transferred onto a glass slide, covered with a coverslip and observed under light microscope (see Section 4.8). At each incubation time, an aliquot of the samples was vigorously vortexed to disassemble the BLSs, subsequently diluted 10-fold in TSB, and plated on TSA for the enumeration of the CFU.

### 4.8. Visualization and Quantification of BLSs

BLSs were examined under an Olympus BX40 microscope with a 40× or 100× oil immersion objective and photographed using Olympus C-5060 camera. BLSs were quantified based on their size in 50 randomly selected microscope fields. Two different operators performed the analysis independently. The size of each BLS was evaluated by establishing a scoring based on the area of the microscope field occupied by the aggregate (1/4, 1/2, 3/4, 1 field). BLS content of a sample was determined dividing the sum of the score values by the number of microscope fields analyzed (i.e., 50) and expressed in percent.

### 4.9. Statistical Analysis

All statistical analyses were performed using one-way ANOVA followed by Tukey–Kramer post-hoc test. A *p*-value less than 0.05 was considered statistically significant. The data are means ± error standard of the mean (SEM) of three independent experiments.

## 5. Conclusions

Overall, the in vitro data obtained in this study demonstrated that the AMP lin-SB056-1 displays a very strong and rapid bactericidal activity against both mucoid and non-mucoid strains of *P. aeruginosa*. The combination of lin-SB056-1 with EDTA results in a potentiated antibiofilm effect against *P. aeruginosa* mature biofilms. The enhancement of the antibiofilm activity could be due to the destabilizing action of EDTA on the biofilm matrix as well as to a direct effect on biofilm-embedded cells. Importantly, in culture conditions mimicking the CF sputum, the combination lin-SB056-1/EDTA showed a significant bactericidal effect towards planktonic cells of *P. aeruginosa* and ability to almost completely inhibit the formation of BLS over a 72 h time-span. Future in vivo studies are planned to fully assess the therapeutic potential of the lin-SB056-1/EDTA combination in the therapy of *P. aeruginosa* infections in CF patients.

## Figures and Tables

**Table 1:** Antibiotic resistance profile of *P. aeruginosa* strains used in the study and their susceptibility to lin-SB056-1 peptide in Sodium Phosphate Buffer (SPB) pH 7.4, 1% Tryptone Soya Broth (TSB)*.*

Strain	Phenotype	Resistance Profile ^1^	MBC ^2^
ATCC 27853	Non-mucoid	None	3.12
PaNM01	Non-mucoid	FOS	3.12
PaNM02	Non-mucoid	FOS	3.12
PaM01	Mucoid	AMC-AZT-CIP-CTX-ERT-LVX-SAM-TIG-TOB	1.56
PaM02	Mucoid	AMK-CIP-FEP-FOS-GEN-LVX-TOB	1.56
PaM03	Mucoid	FOS-LVX-TOB	3.12

^1^ AMC: amoxicillin; AZT: azithromycin; AMK: amikacin; FOS: fosfomycin; CIP: ciprofloxacin; CTX: cefotaxime; ERT: ertapenem; FEP: cefepime; GEN: gentamycin; LVX: levofloxacin; SAM: ampicillin-subactam; TIG: tigecycline; TOB: tobramycin; ^2^ MBC: minimal bactericidal concentrations expressed in μg/mL.

## Supplementary Materials

Supplementary materials can be found at www.mdpi.com/1422-0067/18/9/1994/s1.

## Abbreviations

AMP	Antimicrobial peptide
ANOVA	Analysis of variance
ASM	Artificial sputum medium
BLS	Biofilm-like structure
CF	Cystic fibrosis
CFU	Colony-forming units
EDTA	Ethylenediaminetetraacetic acid
FH	Fully hydrated
LB	Luria bertani broth
LPS	Lipopolysaccharide
LUVs	Large unilamellar vesicles
MBC	Minimum bactericidal concentration
MDR	Multi-drug resistant
PB	Phosphate buffer
PBS	Phosphate buffered saline
POPC	1-Palmitoyl-2-oleoyl-sn-glycero-3-phosphatidylcholine
POPG	1-Palmitoyl-2-oleoyl-sn-glycero-3-phospho-(1′-rac-glycerol)
RF	Rehydrated film
SPB	Sodium phosphate buffer
TSA	Tryptone soya agar
TSB	Tryptone soya broth
