# Supplementary material for: The Semi-Synthetic Peptide Lin-SB056-1 in Combination with EDTA Exerts Strong Antimicrobial and Antibiofilm Activity against Pseudomonas aeruginosa in Conditions Mimicking Cystic Fibrosis Sputum
Source: Int J Mol Sci. 2017 Sep 16;18(9):1994. doi: 10.3390/ijms18091994 (PMC5618643; doi:10.3390/ijms18091994)
Supplement: Supplementary file 1 [file ijms-18-01994-s001.zip › ijms-219682-supplementary-author proofreading-corrected.docx]

**Supplementary Materials: The Semi-Synthetic Peptide lin-SB056-1 in Combination with EDTA Exerts Strong Antimicrobial and Antibiofilm Activity against *Pseudomonas aeruginosa* in Conditions Mimicking Cystic Fibrosis Sputum**

**Giuseppantonio Maisetta, Lucia Grassi, Semih Esin, Ilaria Serra, Mariano A. Scorciapino, Andrea C. Rinaldi and Giovanna Batoni**


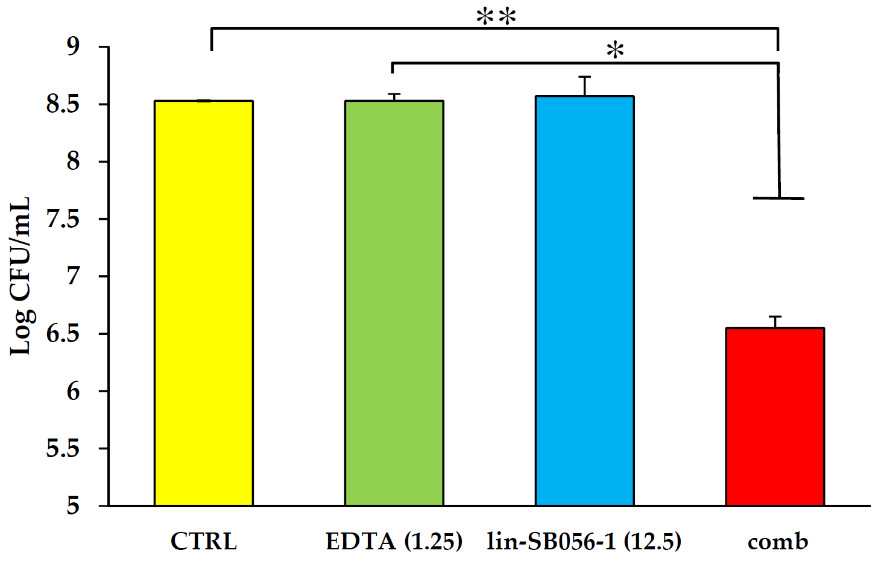


**Figure S1.** Bactericidal activity of lin-SB056-1 used alone and in combination with EDTA against *P. aeruginosa* ATCC 27853 in ASM 80% after 24 h of incubation. The concentrations of lin-SB056-1 and EDTA (reported in parenthesis) are expressed in μg/mL and mM, respectively. Control (CTRL) represents bacteria incubated in the absence of antimicrobial agents; comb: peptide/EDTA combination. Data are reported as mean ± standard error of the mean of three independent experiments. ** *p* < 0.01; * *p* < 0.05 (one way ANOVA followed by Tukey-Kramer post-hoc test).


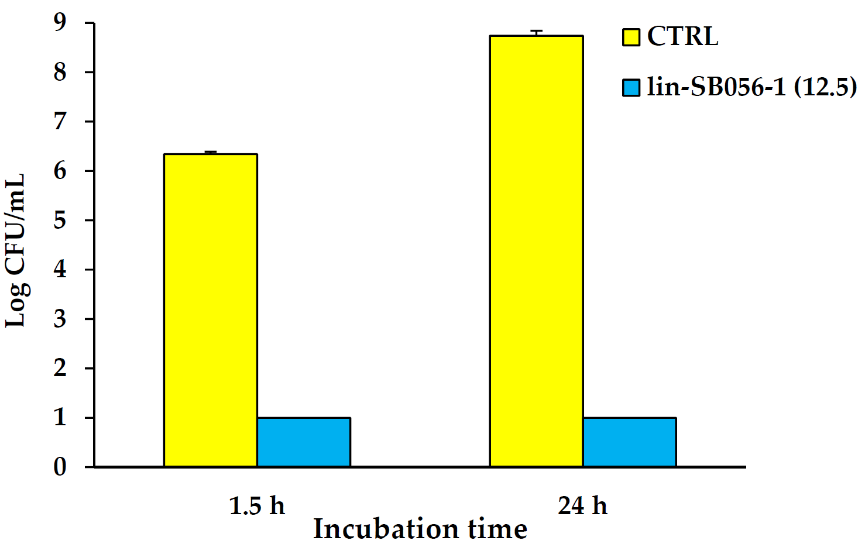


**Figure S2.** Bactericidal activity of lin-SB056-1 in 12.5% human serum against *P. aeruginosa* ATCC 27853**.** The concentrations of lin-SB056-1 (reported in parenthesis) are expressed in μg/mL**.** Control (CTRL) represents bacteria incubated in the absence of peptide.
