# Supplementary figures and images for: The Semi-Synthetic Peptide Lin-SB056-1 in Combination with EDTA Exerts Strong Antimicrobial and Antibiofilm Activity against Pseudomonas aeruginosa in Conditions Mimicking Cystic Fibrosis Sputum
Source: Int J Mol Sci. 2017 Sep 16;18(9):1994. doi: 10.3390/ijms18091994 (PMC5618643; doi:10.3390/ijms18091994)

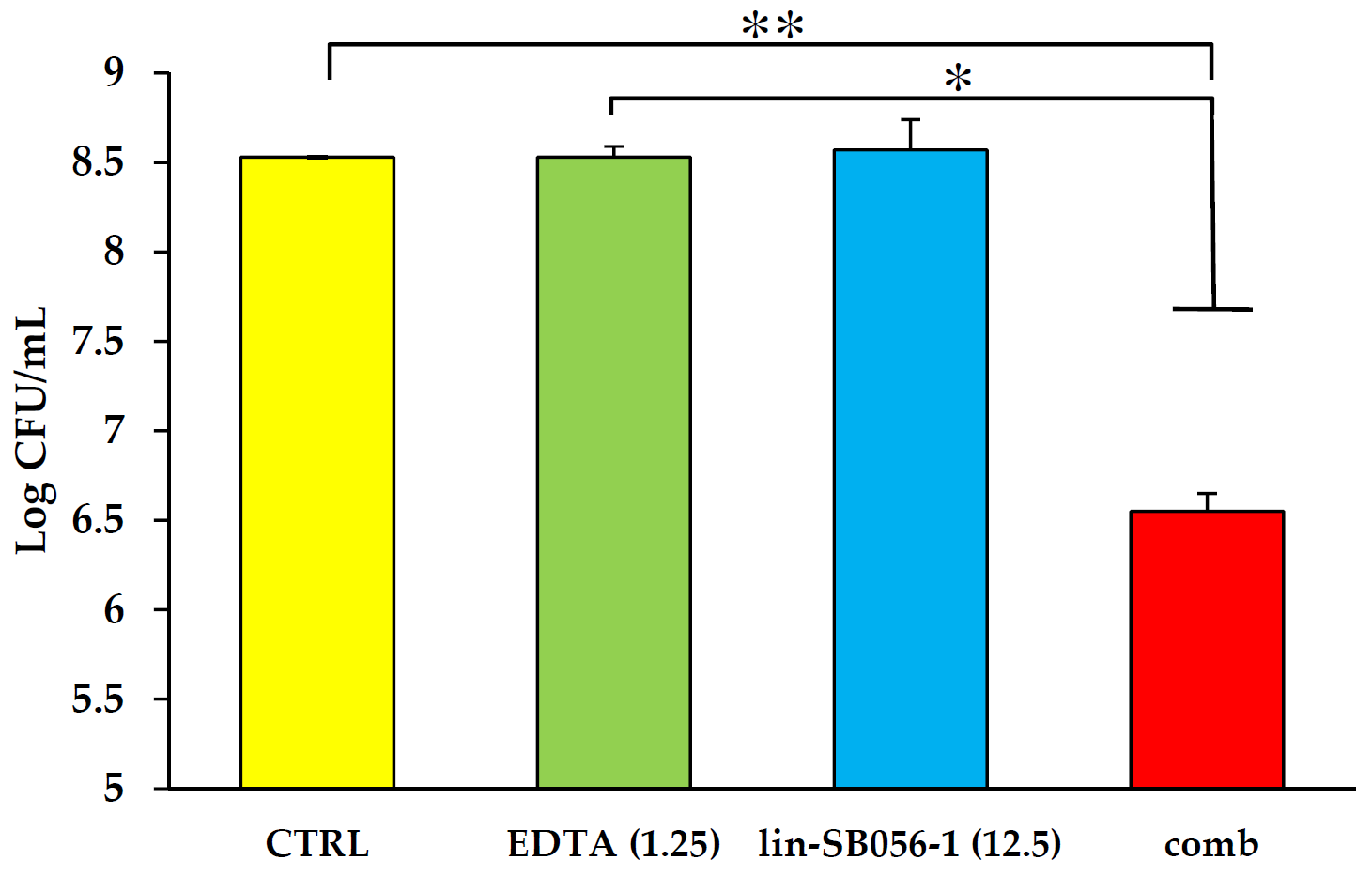

Supplement: Supplementary file 1 [file ijms-18-01994-s001.zip › Figure S1 IJMS-corrected.tif]

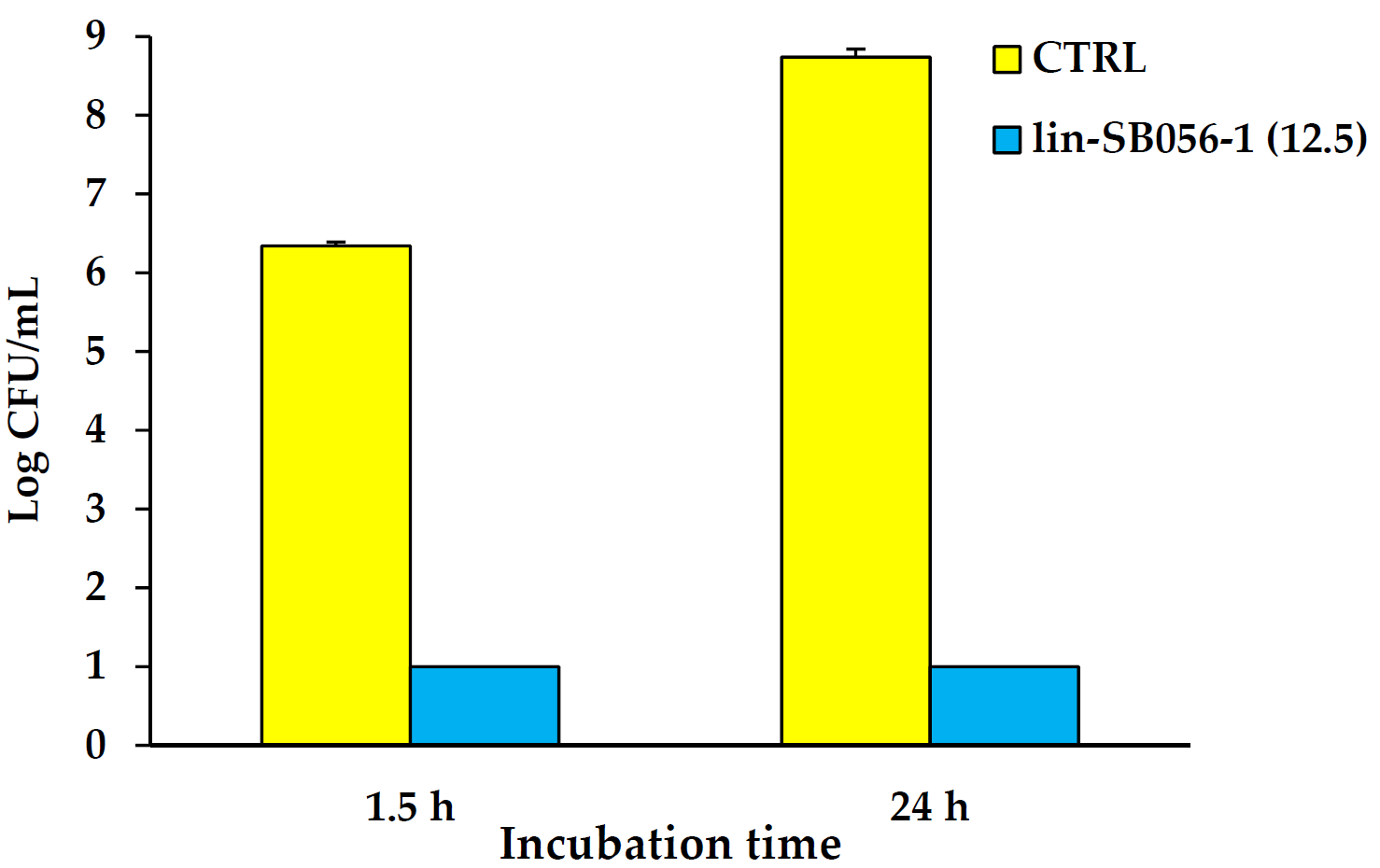

Supplement: Supplementary file 1 [file ijms-18-01994-s001.zip › Figure S2 IJMS-corrected.tif]
